# Epigenetic disruption of cadherin-11 in human cancer metastasis

**DOI:** 10.1002/path.4011

**Published:** 2012-07-26

**Authors:** F Javier Carmona, Alberto Villanueva, August Vidal, Clara Muñoz, Sara Puertas, Rosa M Penin, Montserrat Gomà, Amaia Lujambio, Josep M Piulats, Ricard Mesía, Montse Sánchez-Céspedes, Manel Manós, Enric Condom, Suzanne A Eccles, Manel Esteller

**Affiliations:** 1 Cancer Epigenetics and Biology Programme (PEBC), Bellvitge Biomedical Research Institute (IDIBELL)Barcelona, Spain; 2 Translational Research Laboratory, Catalan Institute of Oncology, Bellvitge Biomedical Research Institute (IDIBELL)Barcelona, Spain; 3 Department of Pathological Anatomy, Bellvitge University Hospital, Bellvitge Biomedical Research Institute (IDIBELL)Barcelona, Spain; 4 Medical Oncology Department, Catalan Institute of Oncology, Bellvitge Biomedical Research Institute (IDIBELL)Barcelona, Spain; 5 Genes and Cancer Laboratory, Cancer Epigenetics and Biology Programme (PEBC), Bellvitge Biomedical Research Institute (IDIBELL)Barcelona, Spain; 6 Cancer Research UK Cancer Therapeutics Unit, Institute of Cancer ResearchSutton, UK; 7 Department of Physiological Sciences II, School of Medicine, University of BarcelonaSpain; 8 Institució Catalana de Recerca i Estudis Avançats (ICREA)Barcelona, Spain

**Keywords:** epigenetics, DNA methylation, cadherin-11, metastasis

## Abstract

Little is known about the molecular events occurring in the metastases of human tumours. Epigenetic alterations are dynamic lesions that change over the natural course of the disease, and so they might play a role in the biology of cancer cells that have departed from the primary tumour. Herein, we have adopted an epigenomic approach to identify some of these changes. Using a DNA methylation microarray platform to compare paired primary tumour and lymph node metastatic cell lines from the same patient, we observed cadherin-11 promoter CpG island hypermethylation as a likely target of the process. We found that *CDH11* DNA methylation-associated transcriptional silencing occurred in the corresponding lymph node metastases of melanoma and head and neck cancer cells but not in the primary tumours. Using *in vitro* and *in vivo* cellular and mouse models for depleted or enhanced *CDH11* activity, we also demonstrated that *CDH11* acts as an inhibitor of tumour growth, motility and dissemination. Most importantly, the study of *CDH11* 5′-CpG island hypermethylation in primary tumours and lymph node metastases of cancer patients showed this epigenetic alteration to be significantly confined to the disseminated cells. Overall, these results indicate the existence of metastasis-specific epigenetic events that might contribute to the progression of the disease. Copyright © 2012 Pathological Society of Great Britain and Ireland. Published by John Wiley & Sons, Ltd.

## Introduction

The colonization of distant organs by tumour cells accounts for 90% of cancer-associated deaths 1. The metastatic process consists of sequential, interrelated steps by which primary tumour cells acquire the capacity to invade the adjacent tissue, enter the systemic circulation, translocate through the vasculature, arrest in distant capillaries, extravasate into the surrounding tissue parenchyma and, finally, proliferate from micrometastases into macroscopic secondary tumours 2–4. Therefore, understanding the mechanisms and players that mediate this process is crucial to predicting, identifying and designing more effective treatments to eradicate it. Within tumour cells, diverse expression programmes influence critical steps in the dissemination of cancer cells, stimulating the invasion of the underlying connective tissue and migration to form distant metastases 5, 6 or acting as metastasis-suppressor factors that encumber metastasis formation without affecting primary tumour development 2–4. The lymphatic route of metastasis is particularly relevant for carcinomas, where regional lymph nodes are often the first organs to develop metastases and may serve as a potential ‘bridgehead’ in further dissemination 7, 8. Most importantly, beyond the first phase of metastasis, which involves the physical translocation of a cancer cell to a distant ‘host’ tissue, we have limited knowledge about the molecular pathways that confer the ability to develop, grow and further disseminate into the distant metastatic site 9.

The dynamic nature of the aberrant epigenetic setting of cancer cells 10, 11 makes it likely that DNA methylation is involved in metastasis formation. Epigenetics is a central mechanism coordinating gene activity in healthy normal cells and, when disrupted in human cancer, impairs the transcriptional equilibrium and contributes to the progression of the disease 10, 11. Among the epigenetic processes, DNA methylation is a subject of intense study, given the direct implications it has for the regulation of gene activity. Indeed, many alterations have been reported in cancer initiation and progression in recent years 10, 11. Many of these occur in the context of 5′-CpG islands. These are regions of dense accumulation of CG dinucleotides and they occur in ≍ 70% of coding genes in mammals. Promoter CpG island hypermethylation events in both coding genes 12–14 and microRNAs 15–17 have been associated with the tumour invasion process and the generation of local distant metastases. However, very little is known about the putative existence of metastasis-specific DNA methylation events occurring in the disseminated cells that might further promote the expansion of the disease.

We used an epigenomic approach in paired primary tumour/lymph node metastasis from the same patients in order to ensure a similar genetic background; we have identified the specific presence of CpG island promoter hypermethylation-associated silencing of the cell-adherence gene Cadherin-11 (*CDH11*) in the disseminated cells. Most importantly, we also show *in vitro* and *in vivo* that *CDH11* functions as a metastasis-tumour suppressor gene.

## Materials and methods

### Human cancer cell lines and tissue samples

Paired-matched primary tumour and lymph node metastasis-derived cell lines SIHN011A and SIHN011B (head and neck) were provided by Dr Suzanne Eccles (Institute of Cancer Research, UK), while IGR39 and IGR37 (melanoma) were purchased from ATCC. The cells were grown in Dulbecco's modified Eagle's medium (DMEM) supplemented with 10% fetal bovine serum (FBS) and penicillin/streptomycin in a 5% CO_2_ atmosphere at 37 °C. Cell lines were transfected by electroporation. SIHN011B cells were transfected with pEGFP–IRES–*CDH11* and selected by adding G418 (Sigma) at 1 mg/ml to the culture medium. SIHN011A cells were transfected with short-hairpin RNA (shRNA) vectors (Origene), specifically targeting the *CDH11* mRNA sequence and Scramble sequence as control (Scb); clones were selected by incubation with puromycin (Sigma) at 1 µg/ml. Primary tumour and lymph node metastases from head and neck tumours and melanoma patients were obtained from the IDIBELL Tissue Biobank, following approval by the institutional ethics committee.

### DNA methylation screening

Briefly, 500 ng genomic DNA were hybridized on the GoldenGate DNA methylation Assay (Illumina) and processed as previously described 12. The results were analysed using GenomeStudio® software. In order to exclude possible sources of technical and biological bias, we filtered the β-values provided by the array according to the associated detection *p* value reported by the assay, selecting a threshold *p* value of 0.01 that allows a clear distinction between reliable and unreliable β-values 12. A biological factor is provided by the fact that one copy of chromosome X is methylated in a woman, so we thereby decided to identify and remove the 44 probes with gender-specific methylation to avoid biases in the subsequent analyses. Subsequently, Student's *t*-tests were conducted to detect differentially methylated CGs between primary and metastatic cell lines. False discovery rate (FDR) values were calculated using the Benjamini–Hochberg method to consider false-positive associations 18. CGs with FDR values < 0.05 were considered as being noteworthy. Unsupervised clustering was carried out with informative probes exhibiting differential methylation between primary tumour and metastatic cells. We filtered out methylation differences according to probe position, aiming to select those genes for which differences in methylation were present upstream of the transcription start site (TSS) and within a canonical CpG island, which is the putative regulatory region of transcriptional activity becoming altered in cancer. Following these criteria, candidate lists resulting from each comparison were crossed, aiming to select common targets of aberrant hypermethylation in the metastatic cell lines for further characterization. As cut-off values, we considered CpG dinucleotides located within 350 bp of the transcription start site, with a DNA methylation difference > 40%, being unmethylated (< 20%) in the primary cells (SIHN011A and IGR39) and hypermethylated (> 60%) in the metastatic counterpart (SIHN011B and IGR37) for at least one of the probes for each of the genes surveyed on the assay.

To validate the findings from the DNA methylation platform, we performed bisulphite genomic sequencing (BS-SEQ) of the promoter region of the genes of interest, as previously described 19. Genomic DNA was converted using the EZ DNA Methylation Gold kit (Zymo Research, Orange, CA, USA). A minimum of eight single clones were interrogated for each sample and the methylation frequency was calculated in each case. DNA methylation in clinical samples was also studied by methylation-specific PCR (MSP), which was performed on bisulphite-treated DNA extracted from formalin-fixed paraffin-embedded (FFPE) samples (primary tumour and lymph node metastases) obtained from the IDIBELL Tissue Biobank, following approval by the corresponding ethics committee. DNA was converted by sodium bisulphite treatment, as previously described. Specific primers were designed using the MethylExpress® program (Applied Biosystems) to examine the methylation status of particular CG sites covering the cadherin-11 promoter region.

### Expression analyses

In order to establish the correlation between the DNA methylation status of the candidate gene and the expression levels, we carried out quantitative RT–PCR (qRT–PCR) and western blot experiments. For qRT–PCR experiments, total RNA was extracted using Trizol® reagent and 2 µg was retrotranscribed using the ThermoScript™ RT–PCR System (Invitrogen). The reaction was carried out using SYBR Green (Applied Biosystems), and *HPRT1* and *GAPDH* were used as housekeeping genes to enable normalization. Expression levels were quantified and compared with those of the corresponding normal control-tissues. For immunoblotting, total protein was extracted using Laemmli reagent, and specific antibodies against *CDH11* (32–1700 Zymed, Invitrogen) and nucleolin (C-23, Santa Cruz) were used. Reactivation treatments with the demethylating agent 5-aza-2′-deoxycytidine (AZA; Sigma) were performed at 2.5 µm for 72 h, and the recovery of transcriptional activity in both metastatic cell lines was quantified by qRT–PCR and western blot. Immunohistochemistry was performed on FFPE tissue, using a monoclonal mouse anti-*CDH11* (MAB1790, R&D Systems) at 1:50 dilution, and stained sections were evaluated by an expert pathologist in a blinded manner.

### *In vitro* proliferation and invasion assays

Cell proliferation was determined by the 3-(4,5-dimethylthiazol-2-yl)-2,5-diphenyltetrazolium bromide (MTT) assay. The proliferation of SIHN011B-*CDH11* #1, #2, Mock and SIHN011A-57/5 and scrambled shRNA samples was quantified for 5–6 days, staining the cells with MTT and measuring the absorbance at 595 nm. Colony formation assay was performed by seeding 1000 cells onto six-well plates and maintaining them on selection media. After 14 days, cells were stained and fixed and colonies > 1 mm diameter were quantified using GeneSync® software. Invasion features of the cells were evaluated using the Chemotaxis Cell Invasion Assay (ECM550, Millipore), following the manufacturer's instructions. After 48 h incubation, cells were fixed and stained with crystal violet and invasive cells were quantified from several biological replicates.

### *In vivo* tumourigenicity and metastasis assays

To measure *in vivo* cell proliferation, SCID mice were subcutaneously injected in the flank with 3 × 10^6^ cells of each cell population tested (*n* = 8). Tumour formation was registered over 21 days and final tumour weights were measured. The development of lymph node metastasis in immunosuppressed 5 week-old mice (*n* = 15) was evaluated by orthotopic injection in the submucosa of the tongue of *CDH11* and empty-vector transfected SIHN011B cells and the animals were maintained over 15 days. Lymph nodes were visualized by injection of 50 µl methylene blue in the base of the tongue and the mice were sacrificed after 20–30 min for pathological examination 20. To study lung metastases, immunosuppressed 5 week-old nude mice were subjected to tail-vein injection in order to evaluate the metastatic potential (*n* = 12). Briefly, aliquots of 1.5 × 10^6^ cells from each cell population were filtered and injected into the animals through the tail vein; the mice were maintained for 30 days before being sacrificed, following the appropriate protocols. The lungs were fixed and haematoxylin and eosin (H&E) staining was performed for histological assessment. The experimental designs were approved by the IDIBELL animal facility committee.

### Statistical analyses

Statistical analyses were performed using GraphPad Prism5. Student's *t*-tests, Fisher's exact tests and Mann–Whitney U-tests were carried out to evaluate differences between groups.

## Results

### A DNA methylation microarray approach identifies cadherin-11 5′-CpG island hypermethylation in lymph node metastasis

To identify genes with putative DNA methylation-associated inactivation in human metastasis, we analysed the DNA methylation profiles of cancer cell lines derived from the primary tumour and the lymph node metastasis of the same patient. The selected paired cancer cell lines were SIHN011A (primary squamous head carcinoma)/SIHN011B (lymph node metastasis) and IGR39 (primary melanoma)/IGR37 (lymph node metastasis). The DNA methylation fingerprints of each sample were obtained using the GoldenGate DNA methylation BeadArray (Illumina) assay 12, 19–21, which studies the CpG methylation status of 1505 CpG sites located from − 1500 to + 500 bp around the transcription start sites of 808 genes. The panel of genes includes oncogenes and tumour-suppressor genes, imprinted genes, genes involved in various signalling pathways, and those responsible for DNA repair, cell cycle control, metastasis, differentiation and apoptosis 12, 21–23. Sixty-nine percent (*n* = 1044) of the 1505 CpG sites studied are located within a canonical CpG island 24, while 31% (*n* = 461) are situated outside CpG islands. All human chromosomes, except the Y chromosome, are represented among the CpG sites analysed. The obtained DNA methylation microarray data are freely available for download from NCBI Gene Expression Omnibus (http://www.ncbi.nlm.nih.gov/geo) under the accession number GSE32250.

**Figure 1:**
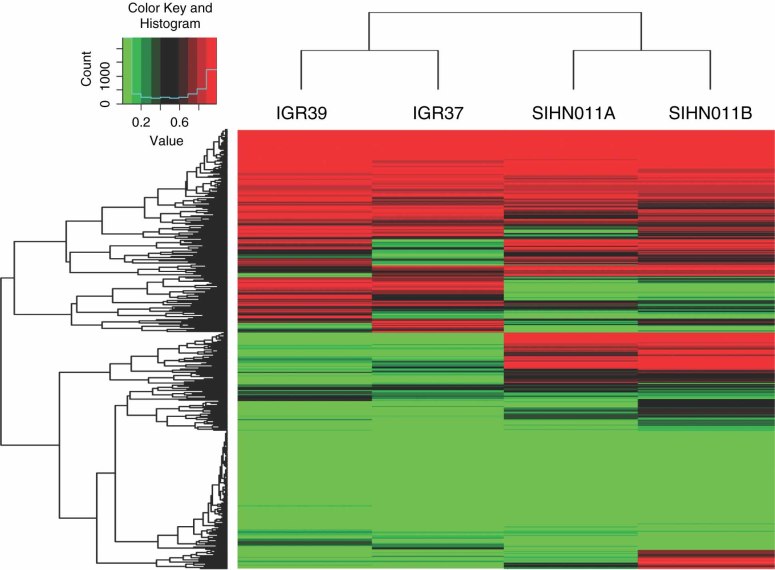
Unsupervised clustering of the DNA methylation profiles obtained from the analyses of the 1505 CpG sites in the same patients' paired primary tumour/metastasis cells in melanoma (IGR39/IGR37) and head and neck cancer (SIHN011A/SIHN011B)

Unsupervised clustering of the DNA methylation profiles obtained from the analyses of the 1505 CpG sites demonstrated that each type of malignancy had its own aberrant DNA methylation profile, head and neck and melanoma samples appearing in distinct branches (Figure 1). Thus, lymph node metastases from the two different tumour types did not cluster together. However, the DNA methylation fingerprint of the metastasis was different to that observed in the primary tumour from whence it came (Figure 1). Although many different CpG sites at different positions and with different CpG methylation levels discriminated the pair primary tumour/metastasis, our objective here was the identification of genes with metastasis-specific epigenetic inactivation. Thus, we set stringent criteria: CpG sites with a > 40% change in CpG methylation level between primary and metastasis; the CpG had to be located within ± 350 bp around the corresponding transcription start site; and the differential CpG methylation primary tumour/metastasis had to occur in both studied tumour type models (head and neck and melanoma). This approach yielded only one target: Cadherin-11 (*CDH11*). The DNA methylation microarray data showed that *CDH11* had a CpG site located in its 5′-CpG island (−173 bp position) that was hypermethylated in the metastatic cell lines IGR37 and SIHN011B but unmethylated in the primary tumour cell lines IGR39 and SIHN011A. Interestingly, roles for *CDH11* in metastasis biology have been proposed 25–28, 35 and thus it was chosen for further characterization.

### DNA methylation-associated transcriptional silencing of *CDH11*

**Figure 2:**
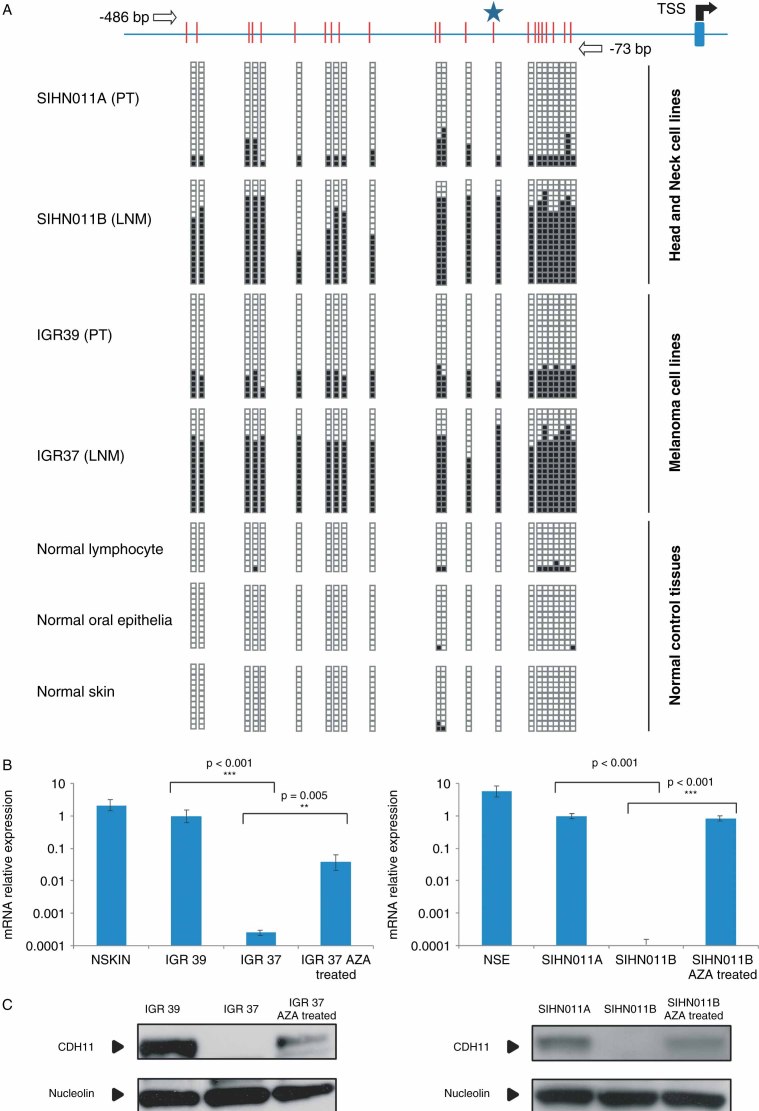
DNA methylation-associated transcriptional silencing of *CDH11*. (A) Bisulphite genomic sequencing of *CDH11* promoter CpG Island. CpG dinucleotides are represented as short vertical lines and the transcriptional start site (TSS) is represented as a long black arrow over a blue stripe. The locations of the bisulphite genomic sequencing primers are indicated by white arrows. A minimum of eight single clones are shown for each sample. Presence of a methylated or unmethylated cytosine is indicated by a black or a white square, respectively. (B) Quantification of *CDH11* mRNA expression levels in IGR39, IGR37, SIHN011A and SIHN011B cell lines and normal control tissues by qRT–PCR. Values are expressed as mean ± SD of three independent experiments, each conducted in triplicate; *p* values were obtained from Student's *t*-tests. (C) Quantification of *CDH11* protein by western blot; nucleolin was used as the loading control

To further demonstrate the presence of *CDH11* 5′-CpG island methylation in metastatic cells, we undertook bisulphite genomic sequencing analyses of multiple clones in the described cancer cell lines. We found dense CpG island hypermethylation in the metastatic cell lines IGR37 and SIHN011B, but mostly an unmethylated CpG island in the primary tumour cell lines IGR39 and SIHN011A (Figure 2A). The presence of a few *CDH11* methylated clones in both primary cancer cell lines might suggest that the metastasis might have arisen from those cells. Bisulphite genomic sequencing of corresponding normal tissues, such as oral epithelial cells and skin, in addition to normal lymphocyte DNA, also showed an unmethylated *CDH11* 5′-CpG island (Figure 2A).

To demonstrate the transcriptional silencing of *CDH11* in metastatic cancer cells in association with the presence of CpG island hypermethylation, we measured *CDH11* mRNA and protein levels by quantitative RT–PCR (qRT–PCR) (Figure 2B) and western blot (Figure 2C), respectively. The expression of *CDH11* was not detectable in the metastatic cell lines IGR37 and SIHN011B, showing CpG island methylation, whilst it was expressed in the unmethylated primary tumour-derived cell lines IGR39 and SIHN011A (Figure 2B, C). The link between DNA methylation and transcriptional silencing was reinforced by the use of a DNA methylation inhibitor; a restoration of *CDH11* mRNA (Figure 2B) and protein (Figure 2C) expression was observed upon treatment with the DNA demethylating agent 5-aza-2′-deoxycytidine in the metastatic cell lines IGR37 and SIHN011B.

### Restoration of *CDH11* expression in metastatic cancer cells decreases growth, motility and dissemination

**Figure 3:**
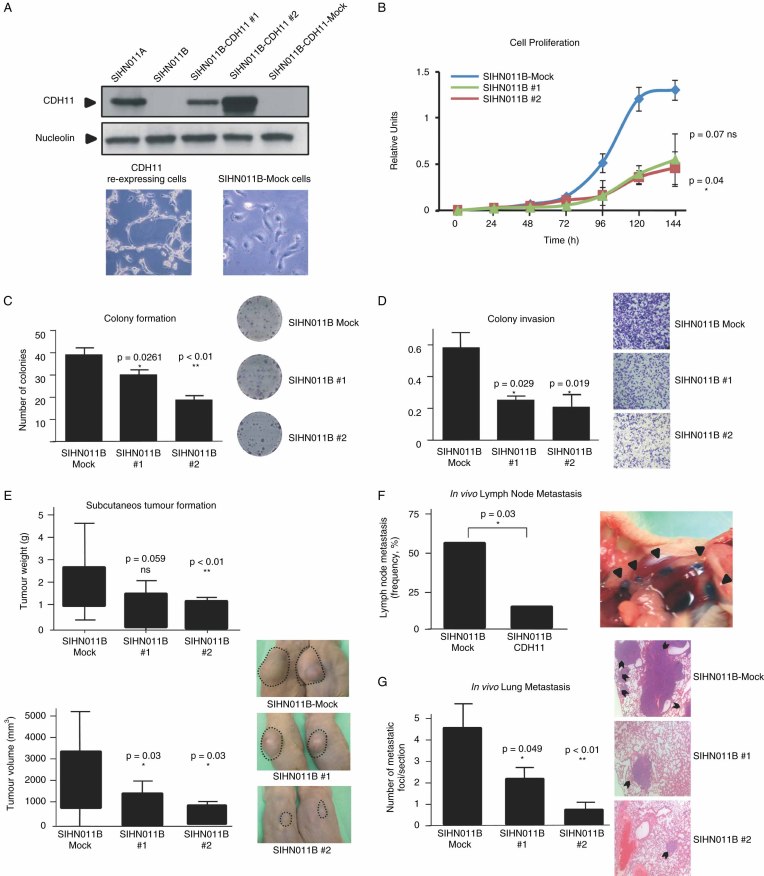
Restoration of *CDH11* expression in metastatic cancer cells reduces growth, motility and dissemination. (A) Full-length cDNA sequence of *CDH11* was cloned in pEGFP–IRES plasmid for stable transfection of cadherin-11 in SIHN011B cells. Two transfected clones (SIHN011B #1 and SIHN011B #2) are shown. (B) Cell proliferation differences between *CDH11*-expressing clones and the corresponding controls determined by the MTT assay and monitored for 6 days; values displayed were obtained from independent experiments with 15 replicates and expressed as mean ± SD; *p* values were obtained from Student's *t*-tests. (C) Colony formation assay. The number of colonies formed 2 weeks after seeding 1000 cells on 35 mm plates and maintained on selection media were quantified and plotted; *p* values were obtained from Student's *t*-tests. (D) Invasiveness assessment, using the Matrigel-coated Boyden chamber assay. The number of cells invading through the membrane was quantified after 48 h of incubation. Images show a representative field of the membrane, and were taken at × 20 magnification; values are expressed as mean ± SD; *p* values were obtained from Student's *t*-tests. (E) Inhibitory effects of *CDH11* expression on tumourigenicity. Head and neck metastatic cells transfected with *CDH11* or empty vector were injected into the flanks of SCID mice. Tumour weight and volume were measured after 21 days. Bars represent the standard error of the mean (SEM); *p* values were obtained from Mann–Whitney U-tests. The photographs show representative cases on day 21 after implantation. (F) The restoration of *CDH11* expression in SIHN011B-transfected cells reduce their capacity to generate lymph node metastasis (detected by methylene blue, right) following injection in the mice tongue submucosa. (G) Metastasis assay through tail vein injection of tumour cells and analysis by H&E staining. Cancer cells with *CDH11* expression (SIHN011B #1 and #2) showed a significant number of metastatic foci; *p* values were obtained from Mann–Whitney U-tests. The photographs show multiple metastatic nodules (black arrows) in SIHN011B-mock tail vein-injected mice

Once we had observed the CpG island hypermethy- lation-associated silencing of *CDH11* in metastatic cancer cells, we sought to demonstrate that the epigenetic inactivation of this gene contributed to metastasis formation. We took two approaches: *in vitro* and *in vivo*. For the former, we stably transfected the metastatic carcinoma cell line SIHN011B, hypermethylated and silenced for *CDH11*, with the pEGFP–IRES–*CDH11* expression vector (see Materials and methods). The efficiency of the transfection was tested by measuring *CDH11* protein levels by western blot (Figure 3A). Upon transfection of *CDH11* in hypermethylated SIHN011B metastasis cells, the cells proved to be significantly less proliferative in the MTT assay (Figure 3B) and had a significantly reduced percentage colony formation density (Figure 3C) in comparison with empty vector-transfected cells. From an invasive feature standpoint, *CDH11*-transfected SIHN011B cells had significantly reduced cell motility capacity, as measured in Matrigel-coated Boyden chambers (Figure 3D).

For the *in vivo* approach, we used tumour and metastasis formation assays in nude mice. First, three million SIHN011B cells transfected with the *CDH11* vector were subcutaneously injected into nude mice. Thirty days after injection, all the mice were killed and tumour weights were measured. Tumours originating from *CDH11*-transfected SIHN011B cells had a significantly lower weight and volume than empty vector-transfected-derived tumours (Figure 3E). Most importantly, we assessed the potentially different capacity of these cells to generate local lymph node metastasis in a head and neck cancer experimental model 20. We observed that *CDH11*-transfected SIHN011B cells orthotopically injected into the submucosa of the tongue of athymic nude mice (*n* = 15/condition) had a significantly lower development of lymph node metastases than empty-vector-transfected SIHN011B cells (Figure 3F). The distant inhibitory dissemination activity of *CDH11* was also measured in athymic mice via tail-vein injection and the analyses of metastasis formation. Whereas numerous metastatic nodules developed in the lung over the 4 weeks following injection of one and half million empty vector-transfected SIHN011B cells (Figure 3G), a significant reduction of metastasis formation was observed with the same number of *CDH11*-transfected SIHN011B cells in the identical period (Figure 3G). These findings are evidence of the role of *CDH11* as a suppressor of tumour dissemination.

### Depletion of *CDH11* expression enhances growth and motility of cancer cells derived from primary tumours

**Figure 4:**
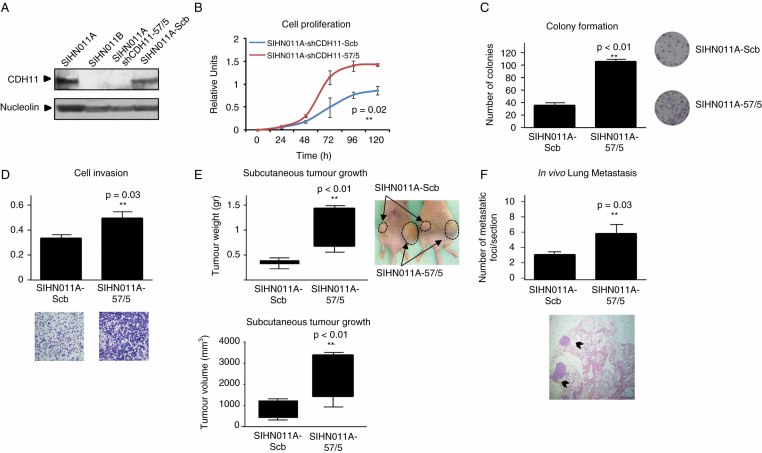
Depletion of *CDH11* expression enhances growth and motility of cancer cells derived from primary tumours. (A) Depletion of *CDH11* expression was achieved through stable *CDH11* shRNA vector transfection (SIHN011A-57/5), compared with the scrambled shRNA-transfected control cells (SIHN011A-Scb). (B) Proliferation rates assessed by the MTT assay were registered over 5 days. Values represented were obtained from independent experiments with 15 replicates and expressed as mean ± SD; *p* value obtained from Student's *t*-test. (C) Colony formation assay. The numbers of colonies formed after 2 weeks were quantified and plotted; a significant increase in the number of colonies was noted in the *CDH11*-knockdown cells; *p* value was obtained from Student's *t*-test. (D) Invasiveness assessment using the Matrigel-coated Boyden chamber assay; the number of cells invading through the membrane was quantified after 48 h of incubation. Cadherin-11-depleted cells SIHN011A-57/5 had significant increased invasive capacities with respect to the control SIHN011A-Scb cells. Values are expressed as mean ± SD; *p* value was obtained by Student's *t*-test. Images show a representative field of the membrane and were taken at × 20 magnification. (E) Enhancing effects of *CDH11* down-regulation on tumourigenicity in athymic nude mice. The head and neck metastatic cells shRNA depleted for *CDH11* and the scrambled shRNA control cells were injected into the flanks of SCID mice. Tumour weight and volume were measured after 21 days. Bars represent SEM; *p* values were obtained from Mann–Whitney U-tests. The photographs show representative cases on day 21 after implantation. (F) Metastasis assay through tail vein injection of tumour cells and analysis by H&E staining. Cancer cells with depleted *CDH11* expression (SIHN011A-57/5) showed a significantly higher number of metastatic foci. The photographs show metastatic nodules (black arrows) in the lung from SIHN011A-57/5 tail vein-injected mice

The restoration of *CDH11* expression in hypermethylated metastatic cells diminished their proliferation and dissemination features. In sharp contrast, we observed that the stable down-regulation of *CDH11* by the short hairpin RNA (shRNA) approach in *CDH11*-expressing and unmethylated primary tumour-derived cells (SIHN011A) had the opposite effects. Following western blot confirmation of *CDH11* depletion by shRNA in SIHN011A cells (Figure 4A), we observed both a significant enhancement of cell proliferation determined by the MTT assay (Figure 4B) and an increase in the density of the colonies formed (Figure 4C) in comparison to scrambled shRNA cells. From the motility aspect, shRNA-mediated depletion of *CDH11* in SIHN011A caused a significant increase in cell invasion, as measured by Matrigel-coated Boyden chambers (Figure 4D). The *in vitro* data were also translated to an *in vivo* setting in athymic nude mice. Three million *CDH11* shRNA-depleted SIHN011A cells were subcutaneously injected into nude mice; 30 days after injection, all the mice were killed and their tumours weighed. Tumours originating from *CDH11* shRNA-down-regulated SIHN011A cells had significantly greater weights and volumes than those derived from scrambled shRNA cells (Figure 4E). We also assessed the inhibitory dissemination activity of *CDH11* in athymic mice via tail-vein injection and the analyses of metastasis formation in the described cells. Whereas numerous metastatic nodules developed in the lung over the 4 weeks following injection of one and half million *CDH11* shRNA vector-transfected cells (SIHN011A-57/5) (Figure 4F), significantly lower metastasis formation was observed with the same number of scrambled shRNA-transfected control cells (SIHN011A-Scb) in the identical period (Figure 4F), supporting the idea that *CDH11* is an inhibitor of cancer progression and dissemination.

### Epigenetic inactivation of *CDH11* occurs preferentially in the lymph node metastases of cancer patients

**Figure 5:**
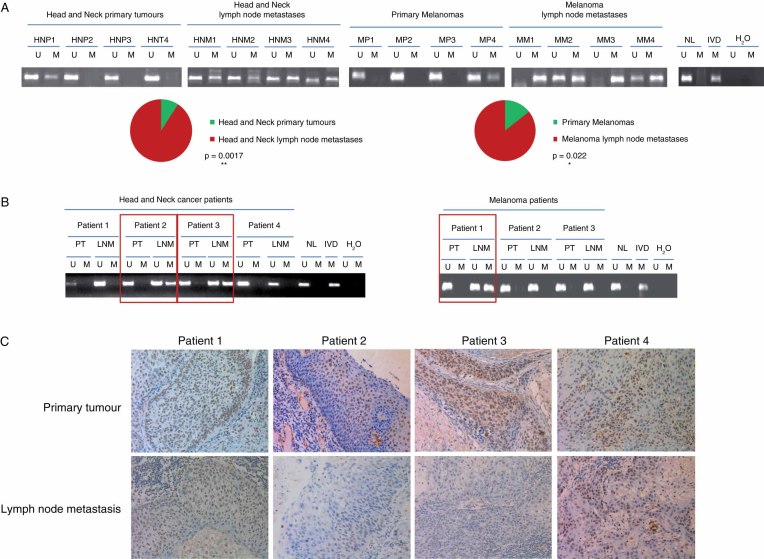
Epigenetic inactivation of *CDH11* occurs preferentially in the lymph node metastases of cancer patients. (A) Methylation-specific PCR was performed on bisulphite-treated DNA extracted from primary tumour and lymph node metastases of head and neck cancer and melanoma patients. The presence of a band under the U or M lanes indicates unmethylated or methylated sequences, respectively. Normal lymphocytes (NLs) and *in vitro* methylated DNA (IVD) are shown as positive controls for the unmethylated and methylated sequences, respectively. The significant enrichment of *CDH11* hypermethylation in lymph node metastasis in comparison to primary tumours is represented in the pie diagrams below; *p* value was obtained from Fisher's exact test. (B) *CDH11* methylation-specific PCR analysis of matched primary tumour/lymph node metastasis from seven patients shows the confinement of *CDH11* hypermethylation in the disseminated samples. (C) Expression of the *CDH11* protein by immunohistochemistry in paired primary tumour/metastasis samples of the four head and neck cancer patients studied in Figure 5B. The presence of *CDH11* CpG island hypermethylation was associated with *CDH11* loss, whereas an unmethylated *CDH11* CpG island was linked to the presence of the *CDH11* protein

The presence of CpG island hypermethylation of *CDH11* was not a specific feature of the metastasis cancer cell lines or an *in vitro* phenomenon. When we analysed a collection of non-cultured tumourigenesis samples from head and neck cancer and melanoma patients, we observed *CDH11* hypermethylation in 19% (13 of 68) and 16% (8 of 51) of cases, respectively. Illustrative examples are shown in Figure 5A. However, the most compelling results concerning the role of *CDH11* epigenetic silencing in tumour dissemination were obtained when the data of *CDH11* hypermethylation were broken down as a function of their sample status: primary tumour or lymph node metastasis. Among the *CDH11* hypermethylated cases, 92% (12 of 13) and 88% (7 of 8) significantly corresponded to lymph node metastasis of head and neck carcinomas and melanomas, respectively; whilst only 8% and 12% of the *CDH11* hypermethylation events occurred in primary tumour samples (Figure 5A) (Fisher's exact test, *p* = 0.0017 and *p* = 0.022, respectively). Most importantly, the pair primary tumour/metastasis from the same patient was available for a subset of seven cases. We observed that *CDH11* hypermethylation occurred preferentially in the lymph node metastasis and not in the primary tumour counterpart (Figure 5B). As occurred with the cell lines, detailed bisulphite genomic sequencing analyses in the primary tumours detected a few *CDH11* hypermethylated clones (see Supplementary Figure 1), from which the metastatic cells might have arisen. Finally, we also analysed the expression of the *CDH11* protein by immunohistochemistry in eight head and neck cancer samples corresponding to the studied paired primary tumour/metastasis of four patients. The presence of *CDH11* CpG island hypermethylation was associated with *CDH11* loss, whereas an unmethylated *CDH11* CpG island was linked to the presence of the *CDH11* protein (Figure 5C). These results highlight the importance of *CDH11* epigenetic silencing in the metastasis microenvironment.

## Discussion

Loss of cellular adhesion is a common feature of human cancer, and the disruption of cell–cell interactions has been recognized as a major contributor to cancer progression. Cadherins are important mediators of cell–cell adhesion, and their involvement in cancer has been widely documented 29. Thereby, the promoter CpG island hypermethylation-associated silencing of *CDH11* in metastatic cells, as a component of this superfamily, is consistent with the hypothesis that epigenetic inactivation of this gene could further enhance cancer dissemination.

Cadherin-11 is an integral membrane protein that binds through the extracellular domain to adjacent *CDH11*-expressing cells. Cadherin-11 is involved in the organization of the synovial lining layer 30 and the differentiation of myofibroblasts 31 and is also an important regulator of neural crest development in *Xenopus laevis* 32. This protein has been studied in relation to its influence on cancer cell behaviour, mainly regarding its metastatic capacity, and a wide range of roles has been assigned to *CDH11*, varying with the tumour types examined. As for other metastasis-suppressor genes, there is a high dependence of suppressive pathways, depending on the tissue context. It is therefore important to consider the cellular make-up, since it has been shown that cadherins might also function differently among distinct cell types 33. For instance, *CDH11* expression in breast and prostate primary tumours—cancers displaying high bone-metastasis tropism—seems to function as a bone-metastasis enhancer, due to the high homologous binding affinity of cadherin-11 for the strongly *CDH11*-expressing osteoblasts, and thus acts to promote invasive phenotypes 34–36. On the other hand, the same protein exerts metastasis-suppressive functions in other cellular contexts. Such is the case for retinoblastoma, where *CDH11* undergoes genomic deletions 26 and its loss correlates with increased invasive phenotypes in cancer cells 35. In a similar manner, *CDH11* expression reduced the lung-metastatic potential and represented a good prognostic factor in osteosarcoma 25, 27. In our models and tumour types (head and neck and melanoma), the functional consequence of *CDH11* loss-of-expression is an enhanced metastatic phenotype, as we have demonstrated experimentally. These findings are also in agreement with the recently reported loss of *CDH11* expression in malignant melanoma as a widespread event 38.

Our findings indicate that *CDH11* behaves in transformed cells like other members of the cadherin superfamily, for which epigenetic disruption of their expression is often associated with increased malignancy. The best-studied is the case of *CDH1* (E-cadherin), whose expression is abolished as part of the ‘cadherin switch’ process, causing the gain of mesenchymal cadherins (typically N- and OB-cadherins), amongst other markers, to the detriment of epithelial E-cadherin 39. Interestingly, a direct interaction between the *CDH11*-cytoplasmatic domain and β-catenin has been demonstrated 40. Furthermore, *CDH11* expression stabilizes β-catenin docking at the cell membrane, providing a link for the decreased proliferative rates that we report, since its binding to *CDH11* reduces Wnt-mediated antiapoptotic signalling 37. Our data suggest that hypermethylation-induced silencing of *CDH11* is a facilitating event for the scattering of tumour cells, by loosening cell–cell contacts and intravasation into blood or lymphatic vessels. In addition, the increased proliferation rates upon *CDH11* inactivation could support further settlements in secondary sites. Finally, it will be worth researching further the putative effect of *CDH11* on cell morphology. Restoration of *CDH11* expression in hypermethylated cells caused a change from a rounded shape to an elongated one (Figure 3A), and it is known that these latter cells are less able to intravasate and withstand shear stresses in circulation 41, further supporting the metastasis tumour-suppressor role of *CDH11*.

*CDH11* CpG island hypermethylation was mostly restricted to the lymph node metastasis. This was shown using clinical samples of two common human malignancies, head and neck carcinomas and melanomas. Head and neck cancer caused 11 000 deaths in the USA in 2010 1 and the survival rates for patients suffering advanced squamous cell head and neck carcinoma were dramatically lower than in those diagnosed at early stages. A similar scenario can be described for melanoma, a malignancy with increasing incidence in the population, refractory to many chemotherapies and with an overall poor survival rate in the population 42. The knowledge that specific epigenetic lesions appear in the metastatic cells from both tumour types, such as *CDH11* hypermethylation, gives us a better understanding of the molecular setting that leads to dissemination and may suggest new biomarkers and therapeutic targets for the advanced forms of the disease.

## Supplementary material on the internet

The following supplementary material may be found in the online version of this article:

Supplementary Figure 1. Bisulphite genomic sequencing of paired primary/metastasis clinical samples.
