# Supplementary material for: Epigenetic disruption of cadherin-11 in human cancer metastasis
Source: J Pathol. 2012 Jul 26;228(2):230–40. doi: 10.1002/path.4011 (PMC3467766; doi:10.1002/path.4011)
Supplement: Supplementary file 1 [file path0228-0230-SD1.pdf]

Figure S1

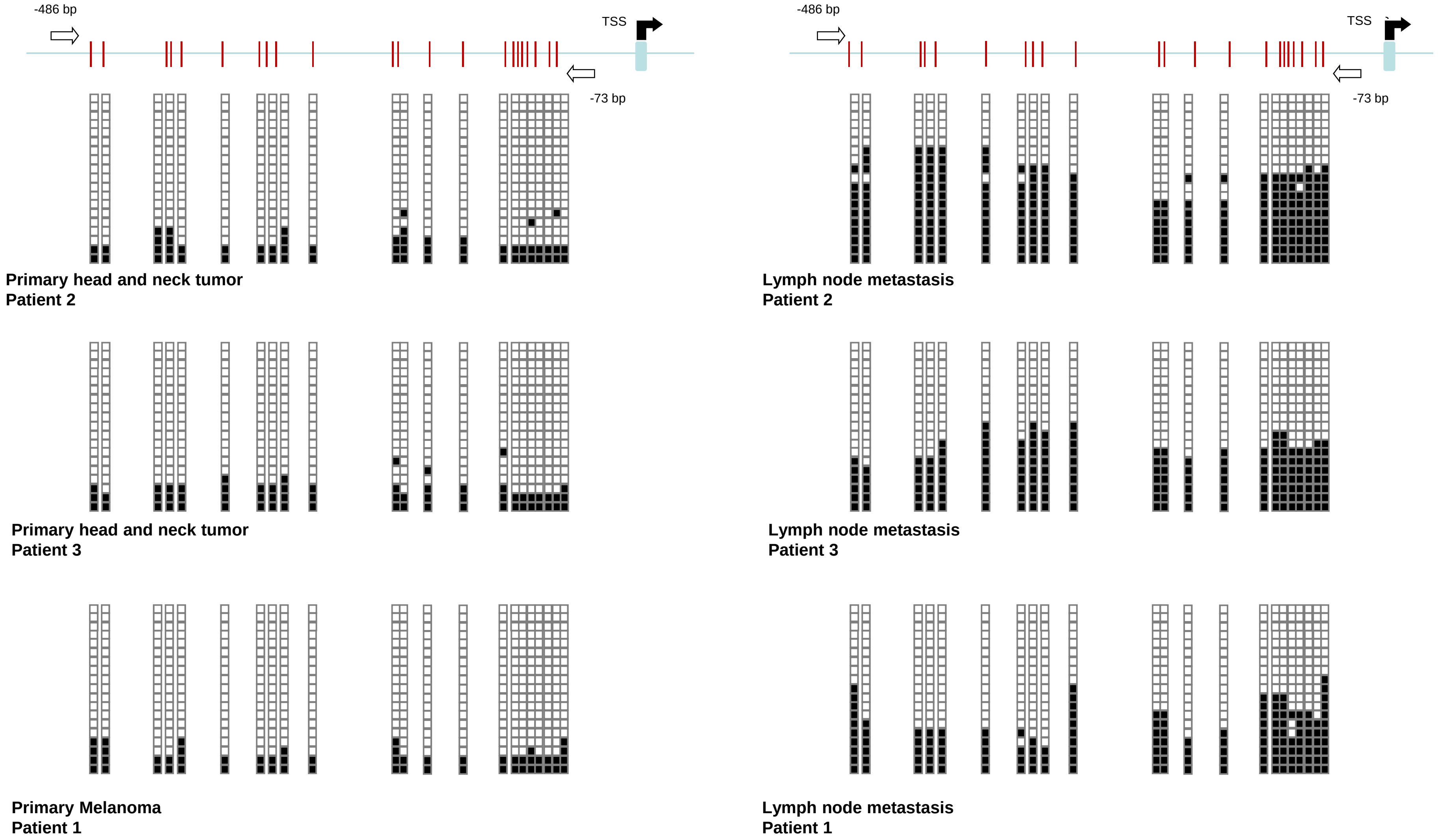

Figure S1. Bisulfite genomic sequencing of CDH11 promoter CpG island in clinical samples from paired primary / lymph node metastasis of head and neck cancer and melanoma patients. CpG dinucleotides are represented as short vertical lines and the transcriptional start site (TSS) is represented as a long black arrow over a blue stripe. The locations of the bisulfite genomic sequencing primers are indicated by white arrows. Nineteen single clones are shown for each sample. Presence of a methylated or unmethylated cytosine is indicated by a black or a white square, respectively.
